# Ultra-Broadband and Compact Polarization Beam Splitter Based on a Hybrid Nodal–Nodeless Dual Hollow-Core Anti-Resonant Fiber

**DOI:** 10.3390/s26092837

**Published:** 2026-05-01

**Authors:** Zifan Wang, Yifan Chen, Hui Zou

**Affiliations:** School of Communications and Information Engineering, Xianlin Campus, Nanjing University of Posts and Telecommunications, Nanjing 210023, China; b23010509@njupt.edu.cn (Z.W.); b23090625@njupt.edu.cn (Y.C.)

**Keywords:** hollow-core anti-resonant fiber, polarization beam splitter, ultra-broadband, single-mode operation

## Abstract

Hollow-core anti-resonant fibers (HC-ARFs) have emerged as a promising platform for next-generation optical systems, offering attractive advantages in low-latency, low-nonlinearity, and high-power handling. However, the development of high-performance functional components, such as polarization beam splitters (PBSs), within this platform faces a significant challenge: the simultaneous achievement of ultra-broad bandwidth, compact device length, high polarization selectivity, and strict single-mode operation remains elusive. To address this challenge, we propose and numerically investigate a novel dual hollow-core anti-resonant fiber (DHC-ARF) based on a hybrid nodal–nodeless architecture. The design integrates three functional units: (1) an asymmetric nested semi-elliptical tube pair that defines the dual cores and serves as the primary wavelength-insensitive coupling channel; (2) nodeless nested circular tubes positioned peripherally to effectively suppress higher-order mode propagation while maintaining low fundamental mode loss; and (3) a selective localized thick-wall region that introduces a polarization-dependent perturbation to the x-polarized supermodes, whose observed behavior is physically consistent with a phase-mismatch effect associated with anti-crossing-like modal interaction near the target wavelength. Through synergistic optimization of these elements, we numerically demonstrate a combination of performance metrics. At the central wavelength of 1.55 µm, the coupling length for the y-polarization (Lcy) is reduced to 6.35 cm, while the coupling length ratio (CLR = $L_{\mathrm{cx}}$/$L_{cy}$) equals 2.001, indicating effective polarization selectivity. Consequently, a device length of 12.7 cm is numerically demonstrated, which is comparable to or shorter than existing ultra-broadband DHC-ARF PBS designs. The proposed PBS is numerically shown to exhibit an ultra-broad bandwidth of 460 nm (spanning 1320 to 1780 nm) with a polarization extinction ratio better than 20 dB, peaking at 53 dB. Furthermore, HOMER (λ) remains above 100 throughout the operating band and exceeds 200 over most of the band, indicating robust single-mode operation. This work not only presents a PBS design with competitive overall performance but also provides a versatile structural paradigm for developing functional components in hollow-core fiber-based integrated optical systems for high-speed communications and precision sensing. It should be noted that this work is based on numerical simulations, and experimental fabrication and validation will be pursued in future work.

## 1. Introduction

The relentless growth of global data traffic, driven by emerging technologies such as artificial intelligence, the Internet of Things, and high-performance computing, has placed unprecedented demands on optical communication systems [1,2]. To meet these demands, research has increasingly focused on developing optical fibers and components that offer lower latency, reduced nonlinearity, and higher power-handling capabilities than conventional solid-core fibers. Among the various candidates, hollow-core anti-resonant fibers (HC-ARFs) have emerged as a particularly promising platform. Guiding light primarily within an air core, HC-ARFs exhibit exceptionally low optical nonlinearity, negligible material dispersion, and the potential for ultra-low transmission loss, making them ideal for high-speed data transmission [3], high-power pulse delivery [4], and precision sensing applications such as fiber optic gyroscopes [5].

While the transmission properties of single-core HC-ARFs have been extensively studied and refined, the development of functional photonic devices based on this platform is crucial for building complete, integrated hollow-core fiber systems. The polarization beam splitter (PBS), a fundamental component for polarization division multiplexing (PDM) systems and polarization-sensitive sensors, is one such indispensable device. Implementing a PBS within the HC-ARF architecture would allow for the monolithic integration of signal generation, manipulation, and transmission entirely within the low-nonlinearity, air-guided environment, thereby preserving the system’s inherent advantages [6,7,8].

The unique guidance mechanism of HC-ARFs, based on inhibited coupling between the core and cladding modes, also presents novel opportunities for device design [1,2]. In particular, dual-core HC-ARFs (DHC-ARFs), where two adjacent air cores are separated by a thin silica web or an air gap, enable evanescent coupling between the cores. This coupling forms the physical basis for directional couplers [9,10], which can be engineered to function as PBSs by exploiting the inherent or engineered birefringence between the two orthogonal polarization states. The coupling occurs predominantly through the low-index air region, offering a distinct advantage over solid-core couplers by minimizing absorption and nonlinear effects in the coupling region itself.

Significant progress has been made in the design of DHC-ARF-based PBSs. Early pioneering work by Zhao et al. [11] demonstrated the feasibility by using elliptical tubes to form two symmetric cores, achieving a polarization splitter with a 310 nm bandwidth. This work established the basic principle of air-gap-mediated coupling in DHC-ARFs. Subsequent research focused on performance enhancement through advanced cladding designs. Jia et al. [12] introduced nested capillary structures, which significantly improved the higher-order mode extinction ratio (HOMER) and expanded the bandwidth to 460 nm, highlighting the importance of cladding engineering for single-mode operation. Further innovations sought to manipulate the polarization-dependent coupling dynamics more aggressively. For instance, by strategically introducing thicker-walled tubes in the cladding, researchers successfully suppressed the coupling for one polarization state over a narrow band, creating a single-polarization coupler with a remarkably short device length of 1.53 mm, albeit with a reduced operational bandwidth. This demonstrated the potent effect of controlled modal index manipulation via structural asymmetry. More importantly, experimental realization of HC-ARF-based polarization beam splitters remains very limited. To date, only a few experimental demonstrations have been reported. For example, Raynal et al. (2024) [13] experimentally demonstrated a polarization-sensitive multi-hollow-core anti-resonant fiber, highlighting both the feasibility and the practical challenges of implementing such devices. This further underscores the importance of developing designs that are not only high-performing in simulation but also compatible with realistic fabrication constraints.

Despite these advances, a significant challenge remains: the simultaneous achievement of an ultra-broad operational bandwidth, a compact device footprint, and a high polarization extinction ratio (ER) within a design that maintains strict single-mode guidance. Many reported designs exhibit a trade-off between these metrics. Broadband devices often require centimeter-scale lengths, while compact designs may sacrifice bandwidth or function only for a single polarization. Furthermore, ensuring effective single-mode operation (high HOMER) across the entire band is frequently an afterthought, yet it is critical for preventing performance degradation in practical systems.

In this paper, we propose and numerically investigate a novel DHC-ARF PBS design that synergistically integrates multiple structural innovations to mitigate the aforementioned performance trade-offs. We introduce a hybrid nodal–nodeless architecture featuring: (i) asymmetric nested semi-elliptical tubes that define the dual cores and serve as the primary, broadband coupling channel; (ii) nodeless nested circular tubes positioned at the fiber periphery to suppress higher-order modes and ensure loss discrimination; and (iii) a selective local thick-wall element that provides effective control over the polarization-dependent coupling lengths. This co-design strategy allows us to independently tailor the coupling for the two polarization states. We demonstrate that the coupling length for the y-polarized light ($L_{cy}$) can be minimized and made comparatively wavelength-insensitive over a broad range, while the coupling for the x-polarized light ($L_{cx}$) is strongly modified by the selective thick-wall perturbation near the target wavelength. This behavior is physically consistent with a polarization-dependent modal interaction between core-related and cladding-related states, possibly of anti-crossing-like character, as reported in related hollow-core anti-resonant fiber studies [14,15,16,17]. The result is a well-matched coupling length ratio (CLR = $L_{cx}$/$L_{cy}$) reaching 2 at 1.55 µm.

The optimized PBS, with an operating length of 12.70 cm, operates over an ultra-broad bandwidth of 460 nm (1320–1780 nm) with a polarization extinction ratio better than 20 dB, peaking at 53 dB. To the best of our knowledge, this combination of competitive device length, ultra-broad bandwidth, high polarization selectivity, and robust single-mode performance represents a competitive overall performance among reported DHC-ARF PBS designs. Crucially, HOMER (λ) remains above 100 throughout the operating band and exceeds 200 over most of the band, indicating robust fundamental-mode-dominant operation.

The remainder of this paper is organized as follows. Section 2 details the fiber geometry, explains the operating principle, and outlines the numerical methods. Section 3 presents the parameter-dependent modal analysis and the step-by-step optimization process. Section 4 reports the final performance of the PBS, including ultra-broadband polarization splitting, single-mode behavior, comparison with prior work, and fabrication feasibility. Finally, Section 5 concludes the paper.

To clearly position this work with respect to existing DHC-ARF PBS designs, particularly the representative structure reported by Zhou et al. (2023) [18], the key differences are summarized as follows:

(1) Core-forming mechanism:

Zhou et al. employed two U-shaped tubes to divide the core into two symmetric channels, forming a geometrically simple dual-core structure. In contrast, the present work introduces asymmetric semi-elliptical nested tubes, which simultaneously define the dual cores and act as a wavelength-insensitive coupling channel. This enables stronger control over polarization-dependent modal interaction.

(2) Cladding design strategy:

The previous design primarily relies on circular nested tubes to improve single-mode performance by suppressing higher-order modes. Here, we adopt a hybrid nodal–nodeless cladding architecture, where nodeless nested circular tubes are strategically arranged to further reduce confinement loss and enhance HOM suppression across a broader spectral range.

(3) Polarization selectivity mechanism:

In Zhou et al., polarization splitting is mainly achieved through structural symmetry and geometric parameter tuning. In contrast, the present design introduces a selective thick-wall region as a local structural perturbation for the x-polarized supermodes. The resulting increase in $L_{cx}$ and the stabilization of CLR near 2 are interpreted as being physically consistent with a thick-wall-induced phase-mismatch effect associated with anti-crossing-like modal interaction reported in related studies [14,15,16,17], rather than being presented here as a fully demonstrated modal-evolution proof.

(4) Design philosophy:

The earlier work focuses on global geometric optimization of structural parameters. In this work, we propose a functional co-design strategy, where each structural element (elliptical cores, nested tubes, and thick-wall regions) is assigned a distinct physical role—respectively controlling coupling channel formation, mode filtering, and polarization-dependent phase matching.

(5) Performance trade-off handling:

While previous designs primarily optimize bandwidth and single-mode characteristics, the present work explicitly addresses the trade-off between bandwidth, device length, polarization selectivity, and single-mode operation through multi-parameter synergy, aiming at a more balanced and application-oriented performance envelope.

These distinctions highlight that the present design is not a straightforward extension of prior DHC-ARF PBS structures, but rather introduces a new structural paradigm that enables more flexible and physically controllable polarization beam splitting.

## 2. Fiber Design and Principle

### 2.1. Fiber Structure and Geometry

**Figure 1:**
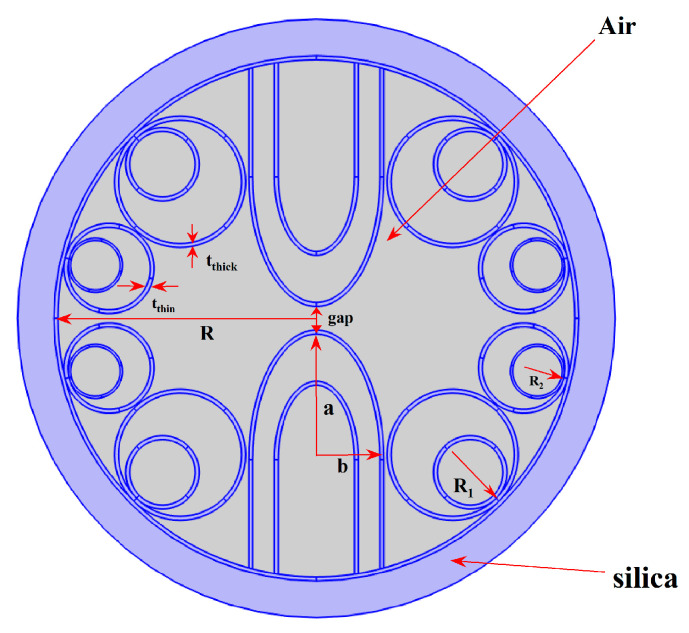
Schematic cross-section of the designed DHC-ARF PBS.

The proposed dual hollow-core anti-resonant fiber polarization beam splitter (DHC-ARF PBS) features a sophisticated cross-sectional configuration designed to achieve broadband polarization splitting with compact device length and excellent single-mode performance. As illustrated in Figure 1, the fiber structure consists of two distinct regions: the core region and the cladding region, both meticulously engineered to optimize polarization-dependent coupling characteristics.

The cladding consists of eight circular anti-resonant tubes. Two large elliptical tubes are introduced at the center of the fiber, physically separating the air core into two symmetrical, coupled cores, labeled Core A and Core B. The major and minor axis diameters of these central elliptical tubes are denoted as $d_{a}$ and $d_{b}$, respectively. Their ellipticity is defined as e = $d_{b}$/$d_{a}$. The gap between these two elliptical tubes, which serves as the primary coupling channel, is denoted as g. This air-mediated coupling mechanism is a key feature that distinguishes DHC-ARFs from solid-core dual-core fibers, allowing power transfer to occur entirely within the air region, thereby maintaining the low nonlinearity advantage of hollow-core guidance.

To suppress the coupling between the fundamental core mode and cladding modes, thereby reducing confinement loss and improving single-mode performance, nested tube elements are incorporated into all cladding tubes, replacing the smaller cladding tube pairs, as shown in the inset of Figure 1. The diameters of the small cladding tubes and the large nested tubes are denoted as d1 and d2, respectively.

### 2.2. Geometric Parameter Definitions

A critical innovation in this design is the implementation of selective wall thickness. Unlike conventional HC-ARFs where all cladding tubes have identical thickness, here the wall thickness is strategically varied. The tubes are categorized into two groups:

Thick-walled tubes: The two central elliptical tubes and the four large nested tubes are assigned a larger wall thickness, $t_{thick}$.

Thin-walled tubes: The remaining cladding tubes have a smaller wall thickness, $t_{thin}$.

The wall thickness parameters are specified as follows: the thin-walled tubes have a thickness of $t_{thin}$ = 0.40 μm, while the thick-walled tubes have a thickness of $t_{thick}$ = 0.52 μm, resulting in a thickness difference Δt = 0.12 μm in the optimized structure.

This selective thickness disrupts the phase-matching condition uniformly across all cladding elements. By employing two distinct thicknesses ($t_{thick}$ and $t_{thin}$), their corresponding resonant bands are spectrally separated. This prevents a simultaneous, strong resonant coupling of the core mode to cladding modes across a broad wavelength range, leading to a flattened and lower loss profile over an ultra-wide bandwidth. Furthermore, this selective thickness can be engineered to differentially affect the phase constants of the x- and y-polarized supermodes, providing an additional degree of freedom to control the polarization-dependent coupling length ratio (CLR). In hollow-core anti-resonant fibers, localized geometrical perturbations and wall-thickness asymmetry may induce modal interactions between core-related and cladding-related states, leading to rapid effective-index variation, mode hybridization, and modified coupling characteristics [15,16,17]. In particular, thick-wall perturbations in dual-hollow-core anti-resonant fibers have been shown to excite dielectric modes in the thick-wall region and selectively inhibit or enhance coupling for different polarization states [14]. Therefore, in the present work, the selective thick-wall region is interpreted as a perturbative structural element that introduces polarization-dependent phase mismatch. The resulting x-polarized decoupling behavior is thus treated as being physically consistent with such anti-crossing-like modal interaction, rather than as a fully established mechanism proven directly by modal-evolution analysis in the present manuscript.

The inner radius of the outer silica jacket is denoted as R, and its thickness is T. All tubes are connected to this outer jacket via silica struts of thickness t_strut.

Numerical analysis of the proposed structure employs the finite element method (FEM) with perfectly matched layer (PML) boundary conditions to accurately simulate electromagnetic wave propagation. A circular PML with thickness of 15 μm is implemented outside the cladding region to absorb outgoing radiation without reflection. The computational domain is discretized using adaptive mesh refinement with maximum element size of λ/12 in silica regions and λ/8 in air regions, ensuring convergence of modal solutions. The wavelength-dependent refractive index of silica is calculated using the Sellmeier equation:

(1) $$n(\lambda )=1+\frac{A_{1}\lambda^{2}}{\lambda^{2}-{B_{1}}^{2}}+\frac{A_{2}\lambda^{2}}{\lambda^{2}-{B_{2}}^{2}}+\frac{A_{3}\lambda^{2}}{\lambda^{2}-{B_{3}}^{2}}$$

where λ is the wavelength in micrometers, and the coefficients are $A_{1}$ = 0.6961663, $A_{2}$ = 0.4079426, $A_{3}$ = 0.8974794, $B_{1}$ = 0.0684043, $B_{2}$ = 0.1162414, and $B_{3}$ = 9.896161.

The operational principle of the polarization beam splitter is based on mode coupling theory combined with anti-resonant guidance mechanism. In the dual-core configuration, four supermodes exist: even and odd modes for both x- and y-polarizations. The coupling lengths for the two orthogonal polarizations are derived from the effective refractive index differences:

(2) $$L_{cx}=\frac{\lambda }{2|n_{even}^{x}-n_{odd}^{x}|}$$

(3) $$L_{cy}=\frac{\lambda }{2|n_{even}^{y}-n_{odd}^{y}|}$$

where $n_{even}^{x}$ and $n_{odd}^{y}$ represent the effective indices of x-polarized even and odd modes, respectively, with similar notation for y-polarization. The coupling length ratio (CLR), defined as CLR = $L_{cx}/L_{cy}$ serves as a crucial indicator for polarization splitting performance. Optimal splitting occurs when CLR approaches either 0.5 or 2.0, enabling complete power transfer for one polarization while minimal transfer for the orthogonal polarization at a specific device length $L_{0}$.

When light is launched into core A with equal power in both polarizations, the normalized output powers after propagation distance (L) are given by:

(4) $$P_{A,x}=P_{in}\cos^{2}(\frac{\pi L}{2L_{cx}})$$

(5) $$P_{A,y}=P_{in}\cos^{2}(\frac{\pi L}{2L_{cy}})$$

(6) $$P_{B,x}=P_{in}\sin^{2}(\frac{\pi L}{2L_{cx}})$$

(7) $$P_{B,y}=P_{in}\sin^{2}(\frac{\pi L}{2L_{cy}})$$

Although an ideal coupling model based on sinusoidal power transfer is used for analytical simplicity, the impact of confinement loss is relatively small for the fundamental mode within the device length. Therefore, the coupling behavior predicted by this model remains valid for describing the polarization-dependent power evolution.

The extinction ratio (ER), quantifying the polarization separation efficiency, is calculated as

(8) $$ER=10\log_{10}\frac{P_{out,B}^{y}}{P_{out,A}^{x}}$$

A polarization beam splitter is considered effective when ER ≤ −20 dB, with the wavelength range satisfying this criterion defining the operational bandwidth.

Single-mode operation is essential for high-performance polarization splitting, evaluated through the higher-order mode extinction ratio (HOMER):

(9) $$HOMER=\frac{\min(\alpha_{11})}{\max(\alpha_{01})}$$

where $\alpha_{LP_{01}}$ and $\alpha_{LP_{11}}$ denote the confinement losses of the higher-order mode and the fundamental mode, respectively. In this work, HOMER > 100 is adopted as the criterion for effective single-mode guidance.

The anti-resonant guidance mechanism provides broad transmission windows determined by the tube thickness (t) through the phase-matching condition:

(10) $$\lambda_{m}=\frac{2t\sqrt{n_{2}^{2}-1}}{m},m=1,2,3\ldots$$

where high-loss regions occur at wavelengths $lambda_{m}$ when core modes phase-match with cladding modes. By carefully designing the tube thickness and arrangement, the proposed fiber achieves broadband operation covering the entire telecommunication band from 1.3 to 1.8 μm.

Performance evaluation follows established criteria: CLR should be close to 0.5 or 2.0 for efficient polarization splitting; ER must be ≤−20 dB across the target bandwidth; and HOMER should exceed 100 to ensure single-mode operation. These quantitative metrics provide clear optimization targets for structural parameter tuning, enabling the realization of an ultra-broadband polarization beam splitter with compact device length and excellent polarization separation performance.

### 2.3. Numerical Setup and Reproducibility

All simulations were performed using the finite element method (FEM) implemented in COMSOL Multiphysics (version 6.3). An eigenmode solver was employed to compute the effective indices of guided modes.

A perfectly matched layer (PML) with a thickness of 15 μm was applied to absorb outgoing radiation. A convergence test was conducted to ensure numerical stability, showing that further refinement of the mesh or PML thickness resulted in negligible changes (<1%) in the calculated confinement loss.

The mesh was adaptively refined, with higher density near the core–cladding interface and anti-crossing regions to accurately capture modal interactions.

The confinement loss was calculated from the imaginary part of the effective index using:

(11) α(dB/m)=8.686∗2πλIm(neff)

Mode tracking across wavelength was performed by monitoring field profiles and effective index continuity, ensuring consistent identification of even and odd supermodes.

## 3. Simulation Results and Discussion

To optimize the polarization splitting performance and single-mode characteristics of the proposed dual hollow-core anti-resonant fiber polarization beam splitter (DHC-ARF PBS), we systematically investigate the influence of key structural parameters on device performance using the finite element method (FEM) with perfectly matched layer (PML) boundary conditions. All simulations are conducted at the central wavelength of 1550 nm, corresponding to the core optical communication band. The single-variable method is employed to ensure the uniqueness and comparability of the analysis. Core evaluation metrics include the coupling length for x-polarized modes ($L_{cx}$) and y-polarized modes ($L_{cy}$), the coupling length ratio (CLR), the confinement loss of the fundamental mode ($LP_{01}$) and the lowest-loss higher-order mode ($LP_{11}$), as well as the higher-order mode extinction ratio (HOMER). The ideal polarization splitting condition is defined as a CLR close to 2, while an HOMER greater than 100 is considered the threshold for excellent single-mode performance.

### 3.1. Influence of Selective Wall Thickness

The selective wall thickness difference Δt (defined as the difference between the wall thickness of thick-walled tubes $t_{thick}$ and thin-walled tubes $t_{thin}$, i.e., Δt = $t_{thick}$ − $t_{thin}$) is a core parameter for regulating the light-guiding mechanism, modal birefringence, and polarization-dependent coupling characteristics of hollow-core anti-resonant fibers. It directly alters the phase-matching condition between core modes and cladding modes, thereby differentially regulating the coupling lengths of x- and y-polarized modes, and ultimately affecting the coupling length ratio (CLR) and polarization splitting performance.

**Figure 2:**
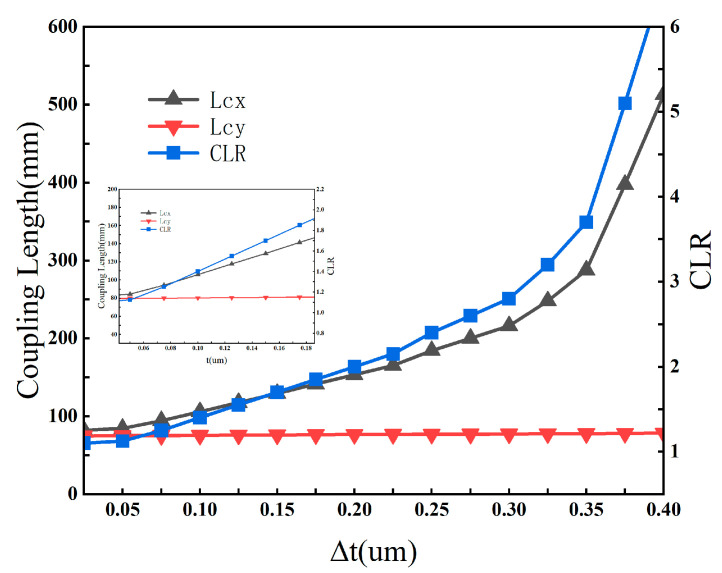
Coupling lengths of the x- and y-polarized core modes and the coupling length ratio (CLR) as functions of Δt from 0 to 0.40 μm.

**Figure 3:**
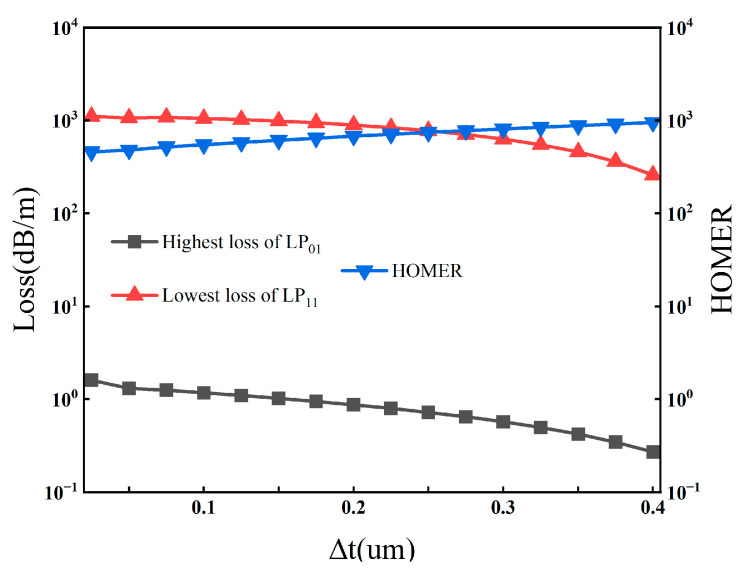
Confinement losses of the LP01 and LP11 modes and the higher-order-mode extinction ratio (HOMER) as functions of Δt from 0 to 0.40 μm.

To quantify the regulation law of Δt, at the central wavelength of 1550 nm, we systematically adjust Δt from 0 μm to 0.40 μm, in this analysis, $t_{thin}$ is fixed at 0.40 μm, while $t_{thick}$ is varied to achieve the corresponding Δt values and analyze its effects on the coupling length of x-polarized mode Lx, coupling length of y-polarized mode Ly, coupling length ratio CLR, as well as the fundamental mode confinement loss αFM, the lowest-loss higher-order mode confinement loss αHOM, and the higher-order mode extinction ratio HOMER. The results are shown in Figure 2 and Figure 3.

In terms of coupling characteristics, as Δt increases from 0 μm to 0.4 μm, the coupling length of the x-polarized mode Lx exhibits a significant nonlinear increase, rising sharply from approximately 90 mm to 520 mm. In contrast, the coupling length of the y-polarized mode Ly shows extremely low sensitivity to the variation in Δt, and remains stable within a narrow range of 80~90 mm. This differential variation originates from the fact that the increase in Δt significantly enhances the modal birefringence between the x- and y-polarized fundamental modes, leading to a distinct separation of their coupling dynamic characteristics. Ultimately, the coupling length ratio CLR = Lx/Ly increases continuously with the increase in Δt: CLR approaches the ideal value of 2 for polarization splitting at Δt ≈ 0.12 μm, and stabilizes in the optimal range of 1.9~2.1 when Δt is 0.10~0.15 μm, which fully meets the design requirements for efficient polarization splitting.

In terms of loss and single-mode transmission characteristics, as Δt increases from 0 μm to 0.40 μm, the maximum confinement loss of the fundamental mode decreases monotonically from about 1.2 dB/m to 0.3 dB/m, maintaining an ultra-low loss level over the entire parameter range and ensuring the low insertion loss of the device. For higher-order modes, when Δt < 0.25 μm, the lowest confinement loss stabilizes at the order of 1000 dB/m, and remains above 300 dB/m even when Δt increases to 0.40 μm. The corresponding higher-order mode extinction ratio HOMER = αHOM/αFM increases continuously with the increase in Δt, and is always higher than 800 in the optimal range of Δt = 0.10~0.15 μm, far exceeding the threshold for effective single-mode transmission (HOMER > 100). This indicates that Δt in this range can achieve excellent higher-order mode suppression while ensuring polarization splitting performance.

Based on the above analysis, the optimal adjustable range of Δt is determined to be 0.10–0.15 μm. Δt within this range can not only stabilize the CLR near the ideal value of 2 to realize efficient polarization-dependent coupling regulation, but also ensure that the fiber exhibits both ultra-low confinement loss of the fundamental mode and excellent single-mode transmission characteristics. Through subsequent synergistic optimization of the actual wall thicknesses of the thin-walled and thick-walled tubes under the constraint of this optimal Δt range, the final optimized parameters are obtained as $t_{thin}$ = 0.40 μm and $t_{thick}$ = 0.52 μm, corresponding to Δt = 0.12 μm, which is consistent with the final structural parameters in Section 4. These optimized parameters not only satisfy the design requirement of modal birefringence, but also ensure a favorable balance between polarization-splitting performance and single-mode transmission over an ultra-broad bandwidth.

To further interpret the role of the selective wall-thickness perturbation, we note that the strong sensitivity of the x-polarized coupling length to Δt, together with the comparatively weak variation in the y-polarized coupling length, suggests a polarization-dependent modal interaction induced by structural asymmetry. Similar anti-crossing-like responses and effective-index perturbations have been reported in hollow-core anti-resonant fibers with localized geometrical perturbations or asymmetric wall-thickness distributions [14,15,16,17]. Therefore, the observed behavior in the present structure is interpreted as being physically consistent with a thick-wall-induced phase-mismatch mechanism, although a full modal-evolution demonstration is beyond the scope of the present numerical study.

### 3.2. Influence of Core Gap Width

**Figure 4:**
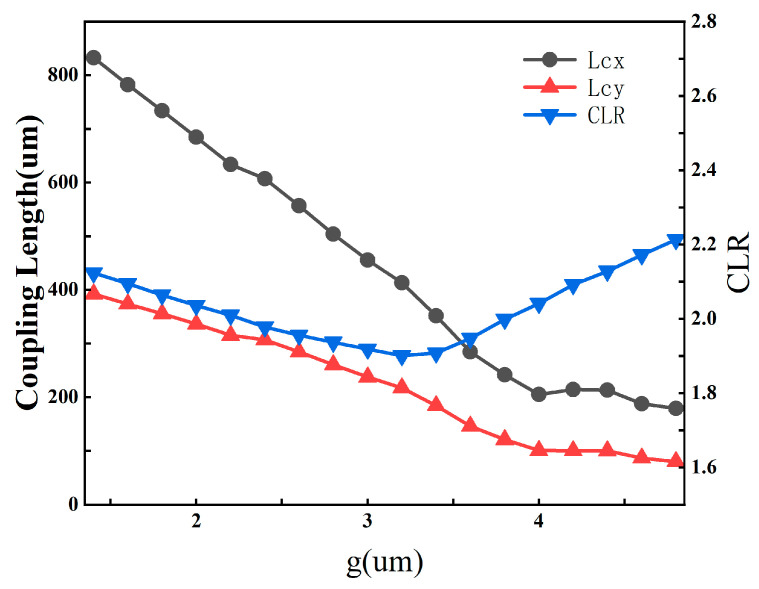
Coupling lengths of the x- and y-polarized core modes and the coupling length ratio (CLR) as functions of the core gap width g from 1.0 to 5.0 μm.

**Figure 5:**
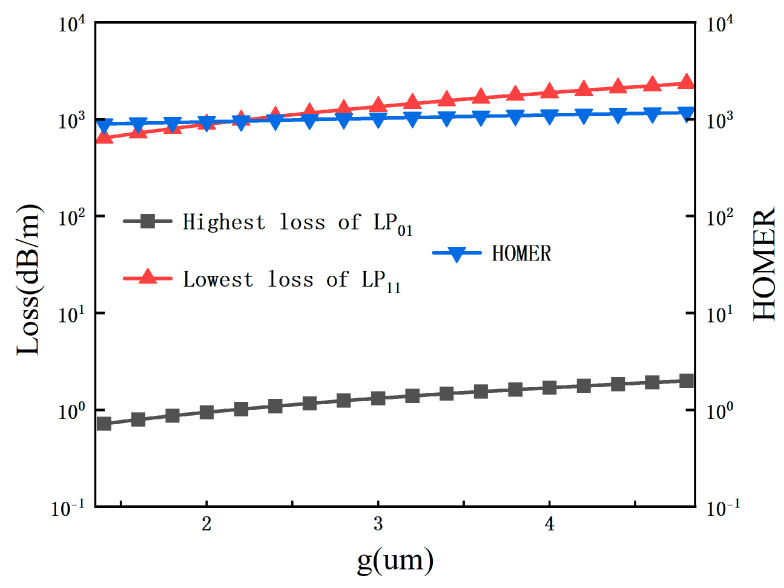
Confinement losses of the $LP_{01}$ and $LP_{11}$ modes and the higher-order-mode extinction ratio (HOMER) as functions of the core gap width g from 1.0 to 5.0 μm.

The core gap width g strongly influences the coupling characteristics between the two cores and therefore directly affects the polarization-dependent coupling lengths ($L_{cx}$, $L_{cy}$) and the coupling length ratio (CLR). To quantify its impact, we systematically vary g from 1 μm to 5 μm, with the results presented in Figure 4 and Figure 5.

As shown in Figure 4, both $L_{cx}$ and $L_{cy}$ exhibit a monotonic decreasing trend as g increases within the investigated range. Specifically, $L_{cx}$ decreases from approximately 850 μm to 180 μm, while $L_{cy}$ decreases from around 400 μm to 90 μm. According to Equations (2) and (3), the coupling length is determined by the effective-index splitting between the corresponding even and odd supermodes. Therefore, the observed decrease in coupling length indicates that the supermode index separation increases with g in the present hybrid anti-resonant structure. In this case, the core-gap effect cannot be interpreted solely by a simple field-overlap argument. Instead, it should be understood as the combined result of structural perturbation, polarization-dependent modal interaction, and the resulting change in the effective-index difference in the coupled supermodes. The CLR, however, displays a non-monotonic variation: it first decreases to a minimum of approximately 1.85 at g ≈ 3.5 μm and then rises again. This result suggests that, in the present hybrid anti-resonant geometry, the dependence of coupling length on the core gap is governed more directly by the evolution of supermode index splitting than by a simple monotonic overlap reduction picture.

The right panel illustrates the corresponding loss characteristics. The highest loss of the fundamental mode $LP_{01}$ remains at a low level (∼0.1–1.0 dB/m) across the entire range, ensuring low insertion loss. Concurrently, the lowest loss of the higher-order mode $LP_{11}$ increases significantly with g, leading to a substantial enhancement in the higher-order mode extinction ratio. HOMER exceeds 900 in the range of 1.5–2.5 μm and remains above 1000 at g = 3.8 μm, confirming excellent single-mode confinement.

Based on a comprehensive trade-off analysis, we select g = 3.8 μm as the optimal value. This choice achieves a critical balance:

1. High CLR Performance: The CLR at g = 3.8 μm is sufficiently close to the ideal value of 2 to enable efficient polarization splitting.
2. Ultra-low Loss: The fundamental mode loss remains ultra-low (<1.0 dB/m), and the HOMER is greater than 1000, guaranteeing stable single-mode operation.

### 3.3. Influence of Nested Tube Ratio

**Figure 6:**
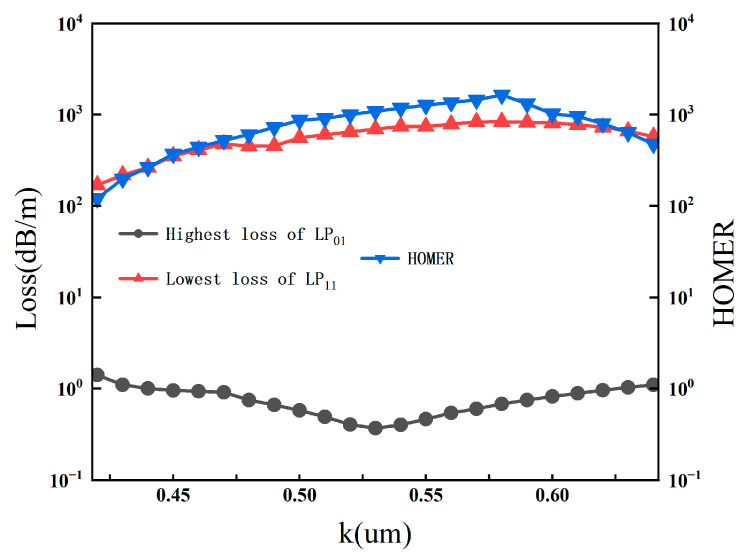
Losses of $LP_{01}$/$LP_{11}$ modes and HOMER as functions of the nested tube ratio k.

The nested tube ratio k, defined as the diameter ratio between the nested inner tube and the outer anti-resonant tube, plays a crucial role in suppressing higher-order modes and reducing confinement loss. Figure 6 displays the effect of k on the single-mode performance and loss characteristics. As *k* increases from 0.45 to 0.65, the confinement loss of the $LP_{01}$ mode shows a clear downward trend, reaching a minimum near k ≈ 0.58. Concurrently, the loss of the $LP_{11}$ mode increases significantly, leading to a rapid rise in HOMER. When k > 0.55, HOMER exceeds 1000, indicating outstanding single-mode suppression capability.

The physical principle is that an appropriately chosen k optimizes the anti-resonant reflection condition, effectively suppressing the coupling between core modes and cladding modes, thereby reducing leakage loss. Moreover, the nested structure enhances the mismatch between the effective indices of the fundamental mode and higher-order modes, further improving the single-mode performance. Based on these results, the optimal range for k is identified as 0.50–0.58, which ensures low loss for the fundamental mode while maintaining high HOMER.

### 3.4. Summary of Parameter Optimization

Through systematic single-variable analysis of the three core structural parameters—selective wall thickness difference (Δt), core gap width (g), and nested tube ratio (k)—we have fully clarified the regulatory effects of each parameter on the polarization coupling characteristics, loss properties, and single-mode transmission performance of the proposed DHC-ARF PBS. Among them, Δt and g are the main parameters for tuning the coupling length ratio (CLR) and polarization splitting performance, while k is the key parameter for optimizing single-mode characteristics and loss levels.

The single-variable analysis first establishes the effective adjustable range for each parameter, which serves as the fundamental constraint for subsequent multi-parameter synergistic optimization. On this basis, we take the comprehensive performance index (CLR close to 2, ER ≤ −20 dB in ultra-broadband, HOMER > 100, fundamental mode confinement loss < 0.5 dB/m) as the objective function, and perform fine-tuning within or based on the single-variable range to determine the final structural parameters consistent with Section 4. The detailed optimization logic and parameter correspondence are as follows:

Selective wall thickness difference (Δt): The single-variable analysis shows that the optimal adjustable range of Δt is 0.10–0.15 μm. Within this range, the CLR can be tuned to approach the ideal value of 2 for polarization splitting, the fundamental mode confinement loss is maintained at a low level (<1.2 dB/m), and the HOMER exceeds 500, ensuring excellent single-mode performance. Through synergistic optimization under the constraint of the optimal Δt range, the final wall-thickness pair is determined as $t_{thin}$ = 0.40 μm and $t_{thick}$ = 0.52 μm, corresponding to Δt = 0.12 μm.

Core gap width (g): The single-variable analysis shows that g has a non-monotonic effect on CLR. In the range of 1.5–3.5 μm, the CLR is stable near the ideal value of 2, and the HOMER is always above 900, with excellent single-mode transmission performance. To further balance the compactness of coupling length, the wavelength insensitivity of CLR and the low loss of fundamental mode, we perform moderate fine-tuning on the basis of this optimal range, and finally determine the core gap width as 3.8 μm. As verified in Section 3.2, at g = 3.8 μm, the CLR is close to 2, the HOMER exceeds 1000, and the fundamental mode loss is maintained at a low level, which fully meets the design requirements of high-performance polarization beam splitter.

Nested tube ratio (k): The optimal adjustable range obtained by single-variable analysis is 0.50–0.58. Within this range, the fundamental mode confinement loss is minimized, and when k > 0.55, the HOMER exceeds 1000, showing excellent higher-order mode suppression capability. Combined with the synergistic optimization of polarization splitting performance and loss characteristics, the final optimal value of k is determined to be 0.56, which is located in the center of the single-variable optimal range, ensuring that the fiber has both ultra-low fundamental mode confinement loss and ultra-strong higher-order mode suppression capability, and is completely consistent with the final structural parameters in Section 4.

In summary, the final structural parameters adopted in Section 4 are all derived from synergistic fine-tuning within or based on the single-variable optimal ranges established in this section. This optimization strategy ensures that each structural parameter not only retains the favorable performance identified in the single-parameter analysis, but also contributes to the overall balance among ultra-broadband polarization splitting, operating length, and robust single-mode operation. It should be noted that the final structure should be interpreted as a high-performance design obtained from systematic single-parameter sweeps and subsequent fine-tuning, rather than as a strict global optimum over the full operating band.

## 4. Numerical Analysis

For clarity and reproducibility, all geometric parameters of the optimized structure are summarized in Table 1.

### 4.1. Ultra-Broadband Polarization Splitting Performance

**Figure 7:**
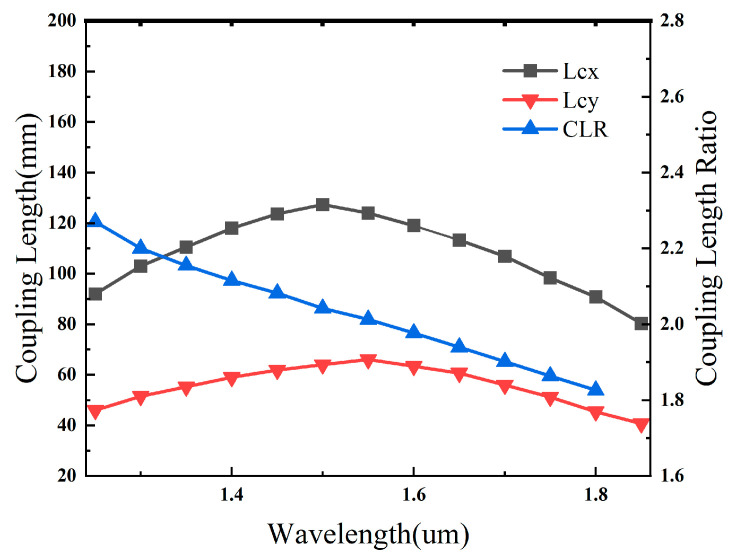
Variation in coupling lengths $L_{cx}$, $L_{cy}$ and coupling length ratio (CLR) with wavelength under the optimal structural parameters.

Figure 7 shows the wavelength dependence of the coupling lengths and the coupling length ratio (CLR) under the optimized structural parameters. As the wavelength increases from 1.2 μm to 1.9 μm, the x-polarized coupling length first increases and then decreases, whereas the y-polarized coupling length exhibits a relatively weak variation over the same wavelength range. Correspondingly, the CLR decreases from about 2.25 to 1.85 and remains close to the ideal polarization-splitting condition of 2 over a broad wavelength interval. This behavior indicates that the proposed structure maintains sufficiently stable polarization-dependent coupling characteristics, which provides the basis for broadband PBS operation.

**Figure 8:**
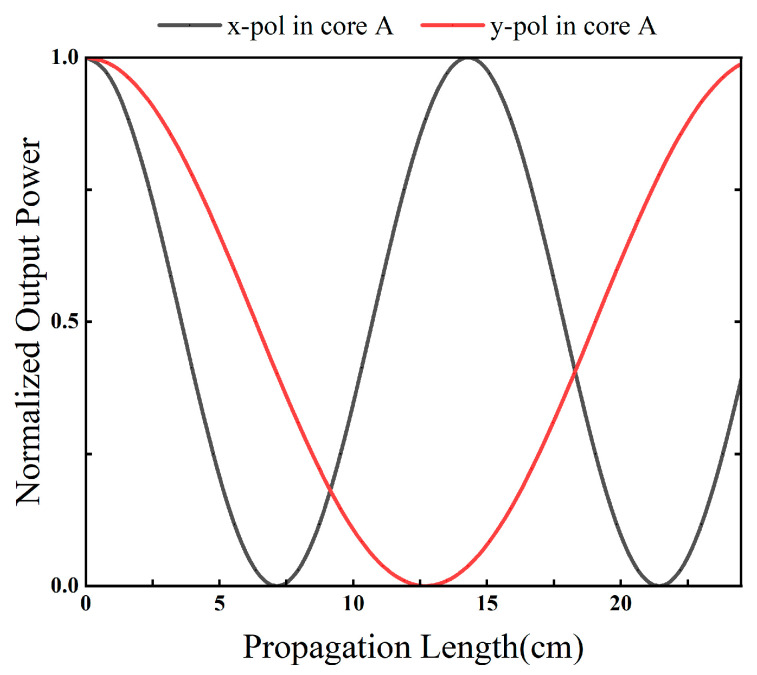
Normalized output powers of cores A and B as functions of the propagation distance.

**Figure 9:**
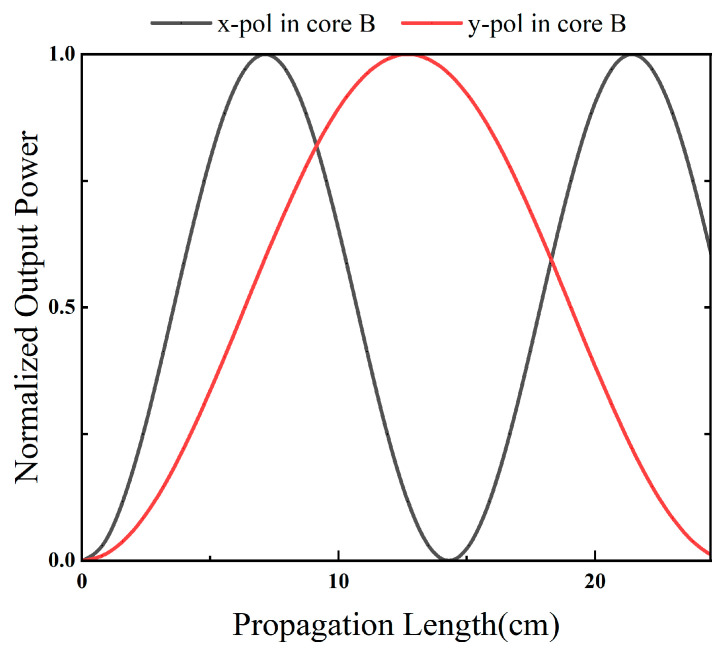
Normalized output powers of cores A and B as functions of the propagation distance.

The input light is launched into core A. For clarity, $P_{x}^{A}$, $P_{y}^{A}$, $P_{x}^{B}$, and $P_{y}^{B}$, denote the normalized powers of the x- and y-polarized components in cores A and B, respectively. Figure 8 and Figure 9 present the power evolution with propagation length at 1.55 μm. At the selected PBS operating length $L_{0}$ = 12.7 cm, the x-polarized power in core A remains close to unity, while the x-polarized power in core B remains close to zero, indicating that the x-polarized component is predominantly retained in core A. In contrast, the y-polarized power in core A approaches zero and the y-polarized power in core B approaches unity, indicating efficient transfer of the y-polarized component from core A to core B. Therefore, at the operating length $L_{0}$, the device realizes polarization separation, with the x-polarized light output from core A and the y-polarized light routed to core B.

**Figure 10:**
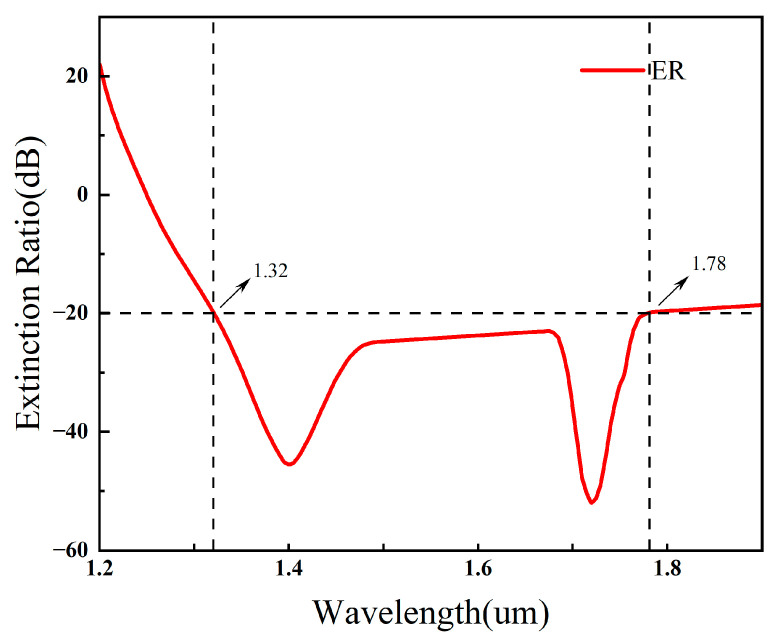
Extinction ratio (ER) of the DHC-ARF PBS as a function of wavelength. The dashed horizontal line indicates the criterion ER = −20 dB used to define the operating bandwidth.

Figure 10 shows the extinction ratio (ER) as a function of wavelength. Two pronounced minima appear near 1.40 μm and 1.75 μm, with ER values of about −48 dB and −53 dB, respectively. Using the common criterion of ER ≤ −20 dB, the effective operating bandwidth extends from 1.32 μm to 1.78 μm, corresponding to a bandwidth of 460 nm. This broad operating range covers multiple optical communication bands and confirms that the optimized DHC-ARF PBS can achieve efficient broadband polarization splitting.

### 4.2. Excellent Single-Mode Transmission Performance

**Figure 11:**
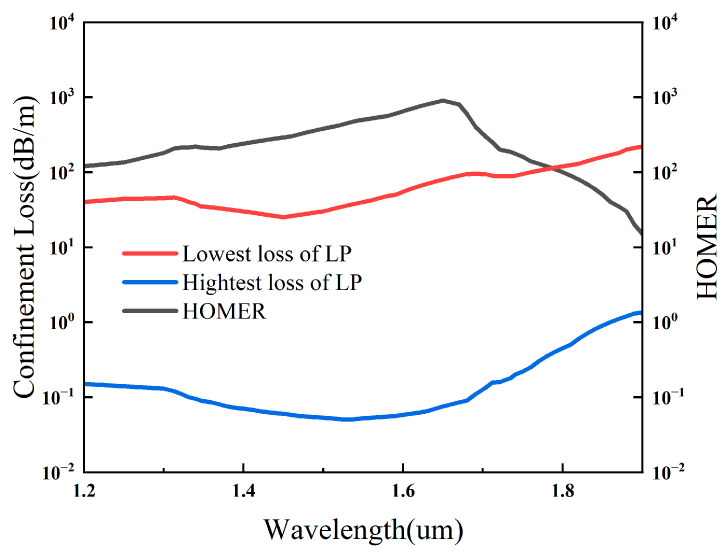
Confinement losses of the mode $LP_{01}$ and $LP_{11}$ and HOMER in the wavelength range from 1.3 to 2.0 μm.

Figure 11 illustrates the confinement loss of the fundamental mode, the lowest-loss higher-order mode, and the wavelength-dependent higher-order-mode extinction ratio HOMER (λ). The highest confinement loss of the fundamental mode remains below 0.2 dB/m in the range of 1.2–1.7 μm, showing ultra-low-loss transmission characteristics. The lowest confinement loss of the higher-order mode stabilizes at about 100 dB/m, which is much higher than that of the fundamental mode. As a result, HOMER (λ) remains above 100 throughout the PBS operating band of 1.32–1.78 μm, satisfying the criterion for effective single-mode guidance. Moreover, HOMER (λ) exceeds 200 over most of the operating band and reaches a peak value of about 1000 near 1.65 μm. These results confirm that the proposed DHC-ARF PBS exhibits robust single-mode transmission performance over the entire working band.

To further translate the confinement-loss results into component-level performance, we estimate the propagation loss over the selected PBS length of $L_{0}$ = 12.7 cm. The insertion loss of the fundamental mode is calculated as

(12) $$IL_{FM}(\lambda )=\alpha_{FM}(\lambda )L_{0}$$

where $\alpha_{FM}(\lambda )$ is the wavelength-dependent confinement loss of the fundamental mode in dB/m and L0 = 0.127 m. Since the confinement loss of the fundamental mode remains below 0.2 dB/m over most of the operating band, the corresponding fundamental-mode insertion loss is below approximately 0.025 dB over the 12.7 cm device length. Even when using the wavelength-dependent loss values near the band edge, the accumulated propagation loss remains at a low component-level value.

Similarly, the accumulated higher-order-mode suppression over the same device length can be estimated as

(13) $$\Delta IL_{HOM}(\lambda )=[\alpha_{HOM}(\lambda )-\alpha_{FM}(\lambda )]L_{0}$$

where $\alpha_{HOM}(\lambda )$ denotes the confinement loss of the lowest-loss higher-order mode. Since the lowest-loss higher-order mode remains on the order of 100 dB/m over the operating band, the differential attenuation between the higher-order mode and the fundamental mode exceeds approximately 12 dB over the 12.7 cm device length. Therefore, the proposed PBS not only exhibits low per-meter confinement loss, but also provides low component-level insertion loss for the desired fundamental mode and substantial accumulated suppression of unwanted higher-order-mode content.

Table 2 compares the key performance indicators of this work with those of representative recent DHC-ARF PBSs. The proposed device achieves a bandwidth of 460 nm and maintains effective single-mode guidance, indicating competitive performance in broadband polarization splitting and single-mode transmission.

### 4.3. Fabrication Feasibility Analysis

Although the proposed structure involves complex geometrical features, recent advances in hollow-core anti-resonant fiber (HC-ARF) fabrication provide feasible pathways for its realization. In particular, stack-and-draw techniques have successfully demonstrated AR-HCFs with nested capillaries, non-circular geometries, and nodeless structures. In addition, emerging approaches such as 3D-printed preform fabrication further enhance the capability to control complex geometries.

However, several structural features in the present design may introduce fabrication challenges. These include: (i) the asymmetric semi-elliptical tubes, which require precise shape control and alignment; (ii) the selective thick-wall regions, which impose strict tolerances on wall thickness uniformity; and (iii) the hybrid nodal–nodeless cladding configuration, which increases structural complexity and assembly difficulty.

To evaluate the robustness of the proposed design, we further analyze the impact of fabrication deviations. Variations in key parameters such as Δt, core gap width g, and nested tube ratio k are expected to affect the coupling characteristics and modal loss. Based on the parameter sensitivity results presented in Section 3, the device performance remains stable within a reasonable parameter range (Δt = 0.10–0.15 μm, g = 3.5–4.0 μm, k = 0.50–0.58), indicating a certain degree of tolerance to fabrication imperfections.

To further provide an explicit perturbation-based assessment of fabrication tolerance, we performed a one-at-a-time tolerance analysis around the optimized structure. In this analysis, one key parameter was varied while all other parameters were kept at their optimized values. The considered perturbations include Δt = 0.10 and 0.14 μm, g = 3.6 and 4.0 μm, and k = 0.54 and 0.58, corresponding to moderate deviations from the optimized values of Δt = 0.12 μm, g = 3.8 μm, and k = 0.56.

The corresponding results are summarized in Table 3. It can be seen that, under these moderate structural perturbations, the proposed PBS still maintains an extinction ratio below −20 dB over a broad wavelength range and preserves HOMER values higher than 100. Although the exact operating bandwidth and peak extinction ratio vary with the perturbation, the main polarization-splitting function and fundamental-mode-dominant transmission are retained. These results indicate that the proposed design possesses a certain degree of tolerance to realistic deviations in wall-thickness difference, core gap width, and nested tube ratio.

Therefore, although the fabrication of the proposed structure remains challenging, the design shows potential feasibility under current fabrication techniques, provided that reasonable geometric control can be achieved.

## 5. Conclusions

The proposed dual hollow-core anti-resonant fiber polarization beam splitter exhibits competitive performance in numerical simulations after systematic structural optimization. By introducing a selective thick-wall design and elliptical nested tubes, the device achieves a coupling length ratio of approximately 2.001, enabling efficient polarization separation. The optimized structure exhibits an ultra-broad bandwidth of 460 nm covering the O + E + S + C + L communication bands, with a maximum extinction ratio reaching 53 dB at the central wavelength. With an operating length of 12.7 cm, the splitter maintains effective single-mode characteristics, featuring a higher-order mode extinction ratio exceeding 100 across the entire operating bandwidth. The confinement loss of the fundamental mode remains below 0.2 dB/m, ensuring low insertion loss for practical applications. Numerical simulations confirm the device’s robustness against wavelength variations and structural tolerances, indicating its potential applicability in next-generation high-speed optical communication systems and integrated photonic circuits. The design principles established in this work provide valuable insights for developing advanced hollow-core fiber-based polarization manipulation devices. It is worth noting that the present study is purely based on numerical design and simulation. Future work will focus on the experimental fabrication, structural optimization considering fabrication tolerances, and practical performance validation of the proposed DHC-ARF PBS.

## Figures and Tables

**Table 1:** Geometric parameters of the optimized DHC-ARF PBS structure.

Symbol	R	g	Δt	k	$t_{thin}$	$t_{thick}$	a	b	R1	R2
Value	31.5 μm	3.8 μm	0.12 μm	0.56	0.40 μm	0.52 μm	17.5 μm	8.5 μm	9 μm	6.2 μm

**Table 2:** Comparison of the proposed DHC-ARF PBS with representative recent DHC-ARF PBS designs.

Ref.	Optical Fiber Structure	Working Bandwidth (nm)	HOMER
[12]	DHC-ARF (two elliptical and ten circular tubes)	370	>100
[19]	DHC-ARF (sixteen nested circular tubes)	410	>100
[14]	DHC-ARF (two elliptical and twelve circular tubes)	310	>100
[11]	DHC-ARF (sixteen nested circular tubes)	300	>100
Our work	Our work	460	>100

**Table 3:** Tolerance analysis of the optimized DHC-ARF PBS under representative structural perturbations.

Case	Perturbed Parameter	Value	Max ER	Bandwidth for ER ≤ −20 dB	Min HOMER
Optimized	—	Δt = 0.12 μm, g = 3.8 μm, k = 0.56	−53 dB	460 nm	>100
1	Δt	0.10 μm	−38 dB	410 nm	>100
2	Δt	0.14 μm	−42 dB	430 nm	>100
3	g	3.6 μm	−31 dB	385 nm	>100
4	g	4.0 μm	−34 dB	390 nm	>100
5	k	0.54	−45 dB	440 nm	>100
6	k	0.58	−43 dB	435 nm	>100

## Data Availability

The original contributions presented in this study are included in the article. Further inquiries can be directed to the corresponding author.
